# Investigation of cationicity and structure of pseudin-2 analogues for enhanced bacterial selectivity and anti-inflammatory activity

**DOI:** 10.1038/s41598-017-01474-0

**Published:** 2017-05-03

**Authors:** Dasom Jeon, Min-Cheol Jeong, Binu Jacob, Jeong Kyu Bang, Eun-Hee Kim, Chaejoon Cheong, In Duk Jung, Yoonkyung Park, Yangmee Kim

**Affiliations:** 1 Department of Bioscience and Biotechnology, Konkuk University, Seoul, 05029 Korea; 2 Division of Magnetic Resonance, Korea Basic Science Institute, Ochang, 28119 Korea; 3 Department of Immunology, Lab of Dendritic Cell Differentiation & Regulation, School of Medicine, Konkuk University, Chungju, 380-701 Korea; 4 Department of Biomedical Science and Research Center for Proteinaceous Materials (RCPM), Chosun University, Gwangju, 61452 Korea

## Abstract

Pseudin-2 (Ps), isolated from the frog *Pseudis paradoxa*, exhibits potent antibacterial activity and cytotoxicity. To develop antimicrobial peptides with anti-inflammatory activity and low cytotoxicity, we designed Ps analogues with Lys substitutions, resulting in elevated amphipathic α-helical structure and cationicity. We further substituted Gly^11^ with Pro (Ps-P analogues) to increase bacterial cell selectivity. Ps analogues retained antimicrobial activity and exhibited reduced cytotoxicity, whereas Ps-P analogues exhibited lower cytotoxicity and antimicrobial activity. Tertiary structures revealed that Ps has a linear α-helix from Leu^2^ to Glu^24^, whereas Ps-P has a bend at Pro^11^ between two short α-helixes. Using various biophysical experiments, we found that Ps analogues produced much higher membrane depolarization than Ps-P analogues, whereas Ps-P analogues may penetrate bacterial cell membranes. Ps and its analogue Ps-K18 exhibited potent anti-inflammatory activity in LPS-stimulated RAW264.7 and mouse dendritic cells via a mechanism involving the Toll-like receptor 4 (TLR4) pathway. These activities may arise from their direct inhibition of the formation of TLR4-MD-2_LPS complex, implying that amphipathic α-helical structure with an optimum balance between enhanced cationicity and hydrophobicity may be essential for their anti-inflammatory activity. The bent structure provided by Pro substitution plays an important role in enhancing bacterial cell selectivity and cell penetration.

## Introduction

The rapid evolution of resistance to commonly used antibiotics in pathogens has necessitated the development of novel types of antimicrobial agents. Host defence peptides represent a new class of such molecules, with qualities that offer promise for clinical success^1, 2^. Antimicrobial peptides (AMPs) are produced by different cells and tissues in a wide range of organisms including invertebrates, plants, and animals^3, 4^. AMPs play important roles in host defence systems and innate immunity. Although the detailed mechanisms are not fully understood, AMP antibiotic activity appears to involve transmembrane pore formation or intracellular killing^4–6^.

Different types of AMPs have been isolated from the skin extracts and/or skin secretions of frogs^7–13^. Magainin is a potent and one of the first AMPs isolated from frog skin^7^, which lead to identification and characterization of many other AMPs from amphibian skin. Pseudins 1–4 are four structurally related AMPs isolated from the skin extract of the paradoxical frog *Pseudis paradoxa* (Pseudidae)^10^. Pseudin-2 (Ps; GLNALKKVFQGIHEAIKLINNHVQ) is the most abundant and potent of the four pseudins. It has been reported that Ps analogues with increased cationicity resulting from replacement of neutral or negatively charged residues with Lys can stimulate insulin release from a BRIN-BD11 clonal β-cell line^11^. Similarly, Lys substitutions are known to improve cell selectivity^14^. However, the anti-inflammatory activities of pseudin have not yet been investigated. In addition, proline substitutions can induce conformational change^15^ and membrane insertion^16, 17^ of AMPs, and can reduce their cytotoxicity^18, 19^. The kink region induced by proline enhances bacterial cell selectivity while retaining the antimicrobial activity of AMPs^20^.

Park *et al*. suggested a pore-forming mechanism for Ps^21^, which is amidated at the C-terminus. Amidation at the C-terminus can affect the efficacy and toxicity of AMPs^22^. Other studies have demonstrated that the membrane interaction abilities of AMPs can be altered by C-terminal amidation^23^. Because naturally occurring Ps is not C-amidated^10^ and the mode of action of non-C-amidated Ps may be different from that of amidated Ps, we set out to investigate the detailed mechanism of action of Ps and its tertiary structure in this study by NMR spectroscopy.

Antimicrobial peptides with anti-inflammatory activity are ideal drug candidates for combating Gram-negative bacterial infection^24^. Since understanding the mechanism by which the peptides exerting the anti-inflammatory activity is important to develop more potent peptides, in this study, for the first time, we investigated the mechanism of Ps for its anti-inflammatory activity. To obtain peptides with higher cell selectivity and anti-inflammatory activity, Lys-substituted Ps analogues were designed. Additionally, Ps analogues with a Pro substitution in place of Gly^11^ (Ps-P analogues) were synthesized. Here, we have studied the anti-inflammatory activities of Ps and its analogues in lipopolysaccharide (LPS) stimulated RAW264.7 cells and mouse dendritic cells and their mode of action based on structure-activity relationships. We investigated the anti-inflammatory pathways of Ps and measured the direct binding to TLR4. Data obtained from biophysical experiments as well as western blot experiments revealed that anti-inflammatory activities of Ps peptide may arise from inhibition of the formation of the TLR4-MD-2_LPS complex by direct binding of the peptides to TLR4. This study provides insight into the mechanism of Ps activity and will thus aid in the development of potent bacterial-selective antimicrobial peptides with anti-inflammatory activity.

## Results

### Peptide design

Cationicity is an integral property of antimicrobial peptides, allowing them to interact with anionic phospholipids and/or LPS in bacterial membranes. Ps has a relatively low positive charge (+2) compared to other well-known antimicrobial peptides such as LL-37 (+6), melittin (+6) or papiliocin (+8). Hydrophobicity influences both the antimicrobial activity and cytotoxicity of antimicrobial peptides. A helical wheel diagram of Ps (Supplementary Fig. S1) shows the amphipathicity of Ps, with hydrophobic residues on the right side and hydrophilic residues on the left side. Because Leu^18^ is located on the boundary between the hydrophilic and hydrophobic sides of the amphipathic α-helix, substitution of Leu^18^ with Lys results in an increase in the hydrophilicity of Ps and increases the cationicity to +3. Therefore, a Ps analogue (Ps-K18) with Lys substituted for Leu^18^ was synthesized. A second Ps analogue (Ps-K14-K18) with Lys substituted for both Glu^14^ and Leu^18^ was also synthesized to further increase the cationicity to +5. Ps-K18 and Ps-K14-K18 are hereafter referred to as Ps series analogues. To decrease Ps cytotoxicity, we substituted Gly^11^ with Pro to create Ps-P, Ps-K18-P, and Ps-K14-K18-P, hereafter referred to as Ps-P series analogues, to disrupt the α-helical structure. The sequences, molecular weights, net charges, hydrophilicities, and retention times of Ps and its analogues are listed in Table 1. Compared to Ps and Ps-P, the analogues with Lys substitution have greater hydrophilicity and shorter retention times.

**Table 1:** Amino acid sequences and properties of the peptides.

Peptide	Sequence	Calculated molecular weight	Measured molecular weight	Net charge	Hydrophilicity	Retention time
Ps	GLNALKKVFQGIHEAIKLINNHVQ	2685.8	2685	+2	−0.22	16.99
Ps-K18	GLNALKKVFQGIHEAIK**K**INNHVQ	2700.9	2700	+3	−0.02	15.51
Ps-K14-K18	GLNALKKVFQGIH**K**AIK**K**INNHVQ	2699.2	2699	+5	−0.02	14.66
Ps-P	GLNALKKVFQ**P**IHEAIKLINNHVQ	2725.4	2725	+2	−0.22	15.30
Ps-K18-P	GLNALKKVFQ**P**IHEAIK**K**INNHVQ	2740.2	2740	+3	−0.02	13.58
Ps-K14-K18-P	GLNALKKVFQ**P**IH**K**AIK**K**INNHVQ	2739.3	2739	+5	−0.02	12.21

Hydrophilicity is the total hydrophilicity (sum of all residue hydrophilicity indices) divided by the number of residues, according to the Hopp and Woods index^54^.

### Resonance assignments and structures of Ps and Ps-P

**Figure 1:**
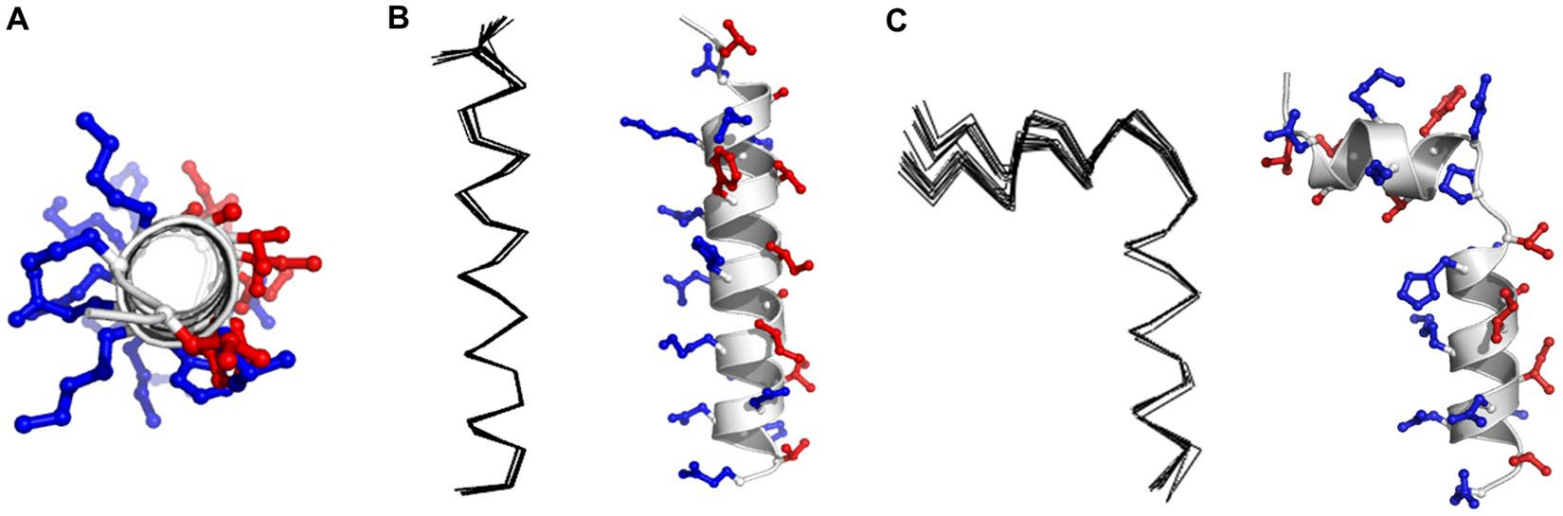
(**A**) Head-on view of the amphipathic N-terminal helix from Leu^2^ to Gln^24^ of Ps. The superpositions of the 20 lowest energy structures were calculated from the NMR data and ribbon diagram of the lowest energy structure for (**B**) Ps and (**C**) Ps-P in 200 mM DPC micelles. The hydrophobic residues are indicated in red, and the hydrophilic residues are shown in blue.

We determined the 3D structures of Ps and Ps-P using NMR spectroscopy by acquiring sequence-specific resonance assignments using double-quantum-filtered correlation spectroscopy (DQF-COSY), total correlation spectroscopy (TOCSY) and nuclear Overhauser effect spectroscopy (NOESY)^25, 26^. Supplementary Fig. S2 shows the NOESY spectra with the sequential assignments for Ps and Ps-P in the NH-CαH region. The chemical shifts of Ps and Ps-P in 200 mM DPC micelles at 303 K referenced to 4,4-dimethyl-4-silapentane-1-sulfonate. To calculate the tertiary structures of Ps and Ps-P, we incorporated experimental restraints such as hydrogen bonding and torsion angle restraints, as well as sequential (|i − j| = 1), medium-range(1 < |i − j| ≤ 5) and long-range(|i − j| > 5) inter-residual distance restraints^26^. Structures with small deviations from the idealized covalent geometry and the experimental restraints (≤0.05 Å for bonds, ≤5° for angles, ≤5° for chirality, ≤0.3 Å for NOE restraints, and ≤3° for torsion angle restraints) were accepted, and we analysed the 20 structures with the lowest energies for each peptide. In Fig. 1A, a head-on view of the amphipathic helix of Ps is shown. We superimposed the 20 lowest-energy structures of Ps (Fig. 1B) and Ps-P (Fig. 1C) over the backbone atoms. Ps has an amphipathic α-helical structure from Leu^2^ to Val^24^ (Fig. 1B). In contrast, Ps-P had two α-helices, one from Leu^2^ to Val^8^ and one from Ile^12^ to Val^23^, and a bend at Pro^11^ in between the two helices (Fig. 1C). The lowest-energy structures of the two peptides in 200 mM DPC micelles are shown in these figures, too. There were no structures with violations >0.5 Å from the NOE distance restraints or >3° from the dihedral angle restraints, and all structures exhibited good covalent geometry. When we superimposed the 20 lowest-energy structures of Ps (from Leu^2^ to Gln^24^) and Ps-P (from Leu^2^ to Val^8^ and Ile^12^ to Val^23^) over the backbone atoms, the root mean squared deviations from the mean structures of Ps were 0.18 ± 0.06 Å for the backbone atoms (N, Cα, C′, O) and 0.77 ± 0.08 Å for all heavy atoms. The root mean squared deviations from the mean structures of Ps-P were 0.34 ± 0.13 Å for the backbone atoms (N, Cα, C′, O) and 0.80 ± 0.09 Å for all heavy atoms (Table S1).

### Circular dichroism (CD) measurements

We analysed the secondary structures of the designed Ps analogues in membrane-like environments using CD measurements (Supplementary Fig. S3). The peptides were dissolved in various membrane-mimicking conditions, and the CD spectra were determined. As shown in Fig. S3, the peptides were unordered in aqueous solution, but they exhibited conformational changes in SDS and DPC micelles. Ps and its analogues adopted a significant degree of α-helical structure in all membrane-mimetic environments, exhibiting characteristic double-negative minima at 205 nm and 220 nm. Analogues that contained a Pro substitution for Gly^11^ showed reduced α-helical structure compared to Ps series peptides (Ps, Ps-K18 and Ps-K14-K18). Lys substitution in Ps-K18 and Ps-K14-K18 increased the α-helical contents compared to Ps. The results from CD and NMR experiments imply that Ps analogues have linear α-helical structures while Ps-P analogues have bent structure in the middle.

### Antimicrobial activity

The antimicrobial activities of the peptides were determined against a representative set of bacterial strains, including four Gram-negative (*Escherichia coli*, *Acinetobacter baumannii*, *Pseudomonas aeruginosa*, and *Salmonella typhimurium*) and three Gram-positive (*Staphylococcus aureus*, *Bacillus subtilis*, and *Staphylococcus epidermidis*) species. Activities of all peptides were compared with that of melittin, a well-known AMP (Table 2). Ps series peptides showed high antibacterial activity, comparable to that of melittin, against Gram-negative bacteria. Ps-K18 and Ps-K14-K18 demonstrated 2- to 4-fold decreases in antibacterial activity against Gram-positive bacteria compared to that of Ps, although their antibacterial activities against Gram-negative bacteria were similar to that of Ps. Introduction of Pro^11^ in Ps, Ps-K18-P and Ps-K14-K18-P resulted in drastic decreases in antimicrobial activity compared to those of Ps series peptides.

**Table 2:** Antimicrobial activities of the peptides against standard bacterial strains.

	MIC^a^ (μM)	Ps	Ps-K18	Ps-K14-K18	Ps-P	Ps-K18-P	Ps-K14-K18-P	Melittin
Gram-negative	*E*. *coli*	1	2	1	8	16	4	4
	*A*. *baumannii*	1	2	2	8	16	8	4
	*P*. *aeruginosa*	2	2	2	16	8	8	4
	*S*. *typhimurium*	2	4	2	8	16	8	8
	Average MIC	1.5	2.5	1.75	10	14	7	5
	MHC^b^	25	100	100	400	400	400	0.4
	Relative Selective Index^c^ (MHC/Average MIC)	16.67	40	57.14	40	28.57	57.14	0.08
Gram-positive	*S*. *aureus*	4	16	8	32	64	32	4
	*B*. *subtilis*	4	8	8	32	64	32	4
	*S*. *epidermidis*	4	8	16	32	64	64	2
	Average MIC	4	10.67	10.67	32	64	42.67	3.33
	MHC	25	100	100	400	400	400	0.4
	Relative Selective Index^c^ (MHC/Average MIC)	6.25	9.37	9.37	12.5	6.25	9.37	0.12

^a^Minimum inhibitory concentrations (MICs) were determined in three independent experiments performed in triplicate with a standard deviation of 14.0%.

^b^The minimal peptide concentration that produces haemolysis.

^c^Ratio of the minimum haemolytic concentration (MHC; μM) over the average of the MIC (μM). Larger values indicate greater cell selectivity.

We also examined the antimicrobial activities of the peptides against different multidrug-resistant bacterial strains, including eight Gram-negative species [*S*. *typhimurium* 8003(R) and 8007(R), *E*. *coli* 1229(R) and 1238(R), *P*. *aeruginosa* 2002(R) and 2095(R), and *A*. *baumannii* 12035(R) and 12036(R)] and three Gram-positive species [*S*. *aureus* 3089(R), 3108(R), and 3126(R)] (Table 3). Ps series peptides showed potent antibacterial activities against multidrug-resistant Gram-negative bacteria and were as effective as Ps and melittin. However, all Ps-P series peptides exhibited much lower activities than Ps series peptides against Gram-negative multi-drug resistant bacteria.

**Table 3:** Antimicrobial activities of the peptides against multidrug-resistant bacterial strains.

	MIC (μM)	Ps	Ps-K18	Ps-K14-K18	Ps-P	Ps-K18-P	Ps-K14-K18-P	Melittin
Gram-negative	MDRST 8003	4	4	4	16	16	16	8
	MDRST 8007	2	4	4	8	16	16	8
	MDREC 1229	2	4	2	8	32	8	4
	MDREC 1238	1	2	2	8	16	8	4
	MDRPA 2002	2	2	2	16	16	16	2
	MDRPA 2095	2	2	4	16	16	16	2
	MDRAB 12035	1	4	2	8	16	16	4
	MDRAB 12036	2	4	2	8	16	8	4
Gram- positive	MRSA 3089	4	16	8	32	64	32	2
	MRSA 3108	4	32	4	32	64	32	2
	MRSA 3126	4	16	4	32	>64	64	2

### Haemolytic activity and cytotoxicity against mammalian cells

**Figure 2:**
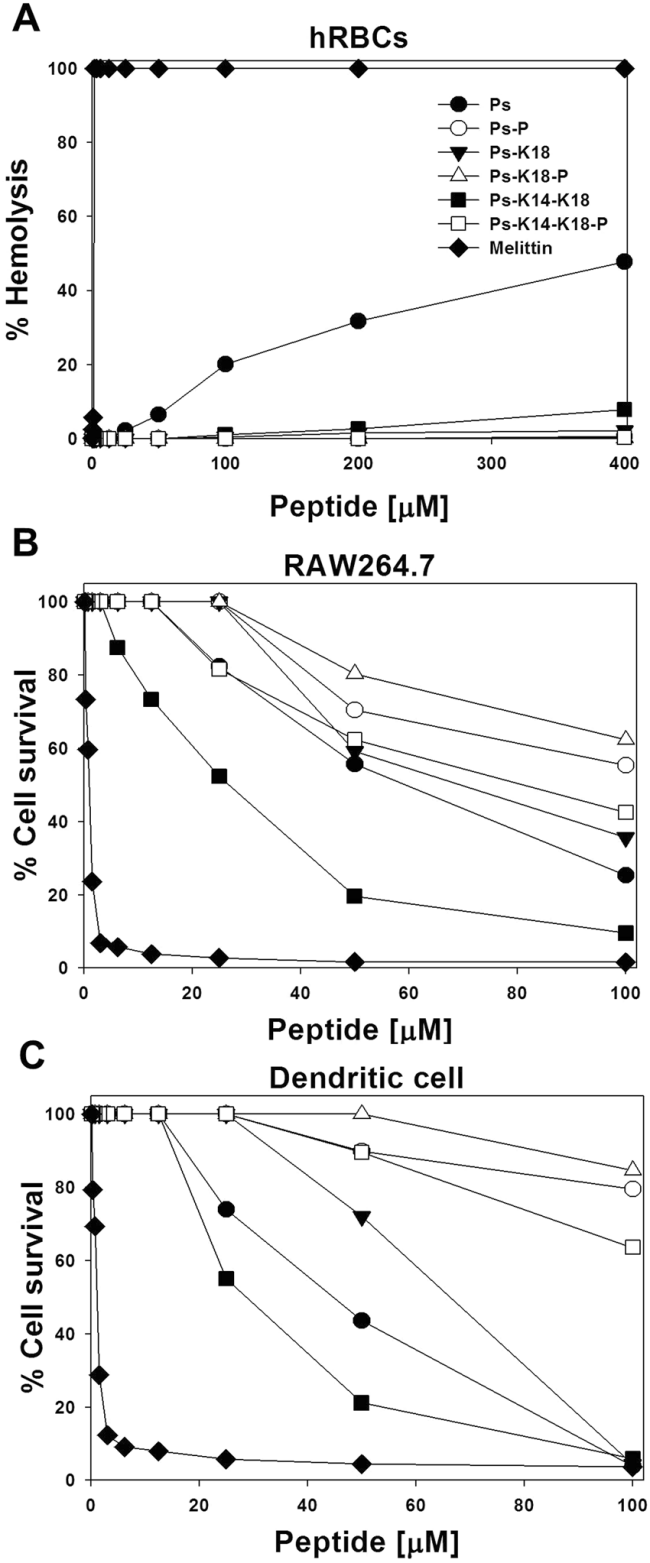
Concentration-dependent toxic activities of Ps and its analogues toward (**A**) human RBCs, (**B**) mouse macrophage-derived RAW264.7 cells and (**C**) mouse bone marrow-derived dendritic cells.

The cytotoxicity of the peptides against mammalian cells was determined by measuring their abilities to cause lysis of human erythrocytes and by determining the cell survival rate of mouse macrophages and bone marrow-derived dendritic cells. Dose-response curves for the haemolytic activities of the peptides are shown in Fig. 2A. Ps resulted in approximately 23% haemolysis at 100 μM while Ps-K18 and Ps-K14-K18 show much lower haemolytic activities (0.5% and 1.1%, respectively) at 100 μM compared to Ps. None of the remaining Ps-P series peptides caused haemolysis at 200 μM. Ps-P, Ps-K18-P and Ps-K14-K18-P showed weak haemolytic activity 0.5%, 0.1% and 0.3% at 400 μM, respectively. Table 2 lists the minimal concentration that produced haemolysis (MHC) against human red blood cells (hRBCs), the average minimum inhibitory concentration (MIC) values for all seven standard bacterial strains tested and the relative selective index (MHC/average MIC). All Ps series analogues exhibited comparable antimicrobial activities to the parent Ps peptide but significantly higher MHC values, making Ps series analogues more selective for Gram-negative bacteria. The antibacterial activities of Ps-P series analogues were several folds lower than that of Ps, but the peptides did not induce haemolysis even at 200 μM concentration tested, resulting in relative selective indexes comparable to that of Ps.

The survival rates of peptide-treated RAW264.7 macrophage cells and mouse bone marrow-derived dendritic cells were determined using an MTT assay, and the results are shown in Fig. 2B,C. At 50 μM, Ps application resulted in 56% and 44% survival of RAW264.7 and dendritic cells, respectively. Ps-K18, with its increase in net charge, resulted in much higher cell survival rates than Ps. However, despite its even higher net charge, Ps-K14-K18 resulted in lower cell survival than that of Ps and Ps-K18, implying that the additional Lys substitution increased its cell cytotoxicity. Similar results of increasing charge beyond an optimum value have toxic effect on mammalian cells had reported previously. Dathe *et al*. had shown the effect of charge on the induced haemolysis of magainin II derived peptides^27^. In their study, increasing net charge up to +5 had resulted in improved activity, but haemolysis has drastically increased when charge is beyond +5. All Pro^11^-substituted analogues resulted in higher cell survival than Ps series peptides, with the greatest survival observed with Ps-K18-P, followed by Ps-P and then Ps-K14-K18-P.

### Dye leakage from model membranes

**Figure 3:**
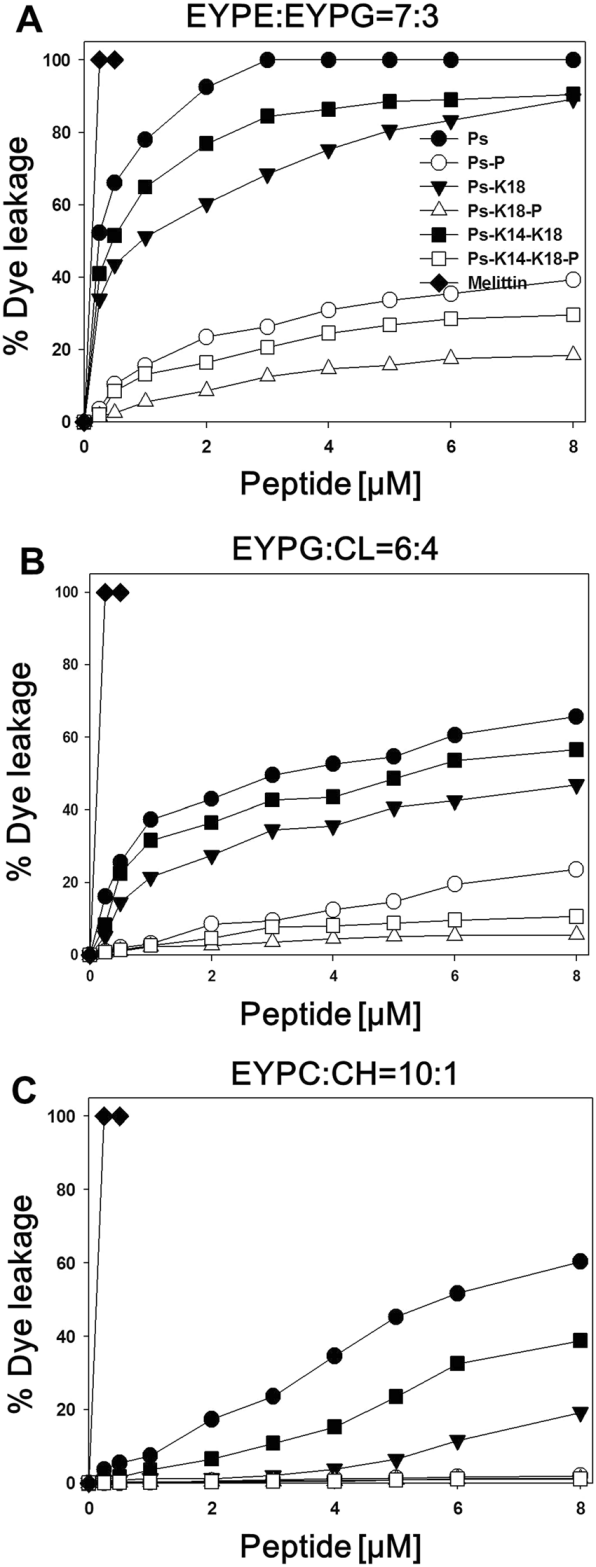
Concentration-response curves of calcein leakage induced by the peptides from (**A**) egg yolk phosphatidylethanolamine (EYPE)/egg yolk phosphatidylglycerol (EYPG) (7:3, w/w), (**B**) EYPG/cardiolipin (CL) (6:4, w/w) and (**C**) EYPC/cholesterol (CH) large unilamellar vesicles (LUVs; 10:1, w/w).

To investigate the mechanism of action of the peptides, we measured the membrane permeabilization abilities of Ps and Ps-P analogues by measuring the release of calcein, a fluorescent marker, from large unilamellar vesicles (LUVs) with lipid compositions that mimic the membranes of Gram-negative bacteria, Gram-positive bacteria and human erythrocytes. We used LUVs with a 7:3 (w/w) ratio of EYPE/EYPG to mimic the membrane of Gram-negative *E*. *coli*, those with a 6:4 (w/w) ratio of EYPG/CL to mimic that of Gram-positive *S*. *aureus*, and those with a 10:1 (w/w) ratio of EYPC/CH to mimic mammalian cell membranes. Figure 3 shows the concentration-response curves of peptide-induced calcein release. The relative abilities of the Ps series peptides to induce strong leakage from EYPE/EYPG vesicles coincide well with the measured high antibacterial activities of the Ps series peptides against Gram-negative bacteria. Moreover, the relative abilities of peptides to induce leakage from EYPG/CL vesicles are consistent with the relative antibacterial activities against Gram-positive bacteria. Ps was highly effective at permeabilizing all membranes, whereas Ps-K18 and Ps-K14-K18 were less effective, implying that hydrophobicity is important for permeabilization ability. However, Ps, Ps-K18 and Ps-K14-K18 were much more effective at permeabilizing vesicles than Ps-P, Ps-K18-P and Ps-K14-K18-P. Notably, Ps-P, Ps-K18-P and Ps-K14-K18-P did not permeabilize neutral EYPC/CH vesicles mimicking mammalian cell membranes, implying that they are highly bacterial cell-selective. The reduced permeabilization abilities of Ps-P, Ps-K18-P and Ps-K14-K18-P compared to Ps, Ps-K18 and Ps-K14-K18 implies a different mechanism of action for Ps-P peptides. Our results showed that the relative abilities of the peptides to induce leakage from zwitterionic vesicles are in agreement with their relative haemolytic activities and cytotoxicities. Ps-K18 shows much less permeabilization abilities against neutral vesicles compared to Ps and Ps-K14-K18, implying that Ps-K18 has high bacterial cell-selectivity. Since the relative selective index of Ps-K18 and Ps-K14-K18 are the same while Ps-K18 has much lower cell cytotoxicity compared to Ps-K14-K18, we selected Ps-K18 as a candidate of potent peptide antibiotics.

### Membrane disruption against *E*. *coli* and *S*. *aureus*

**Figure 4:**
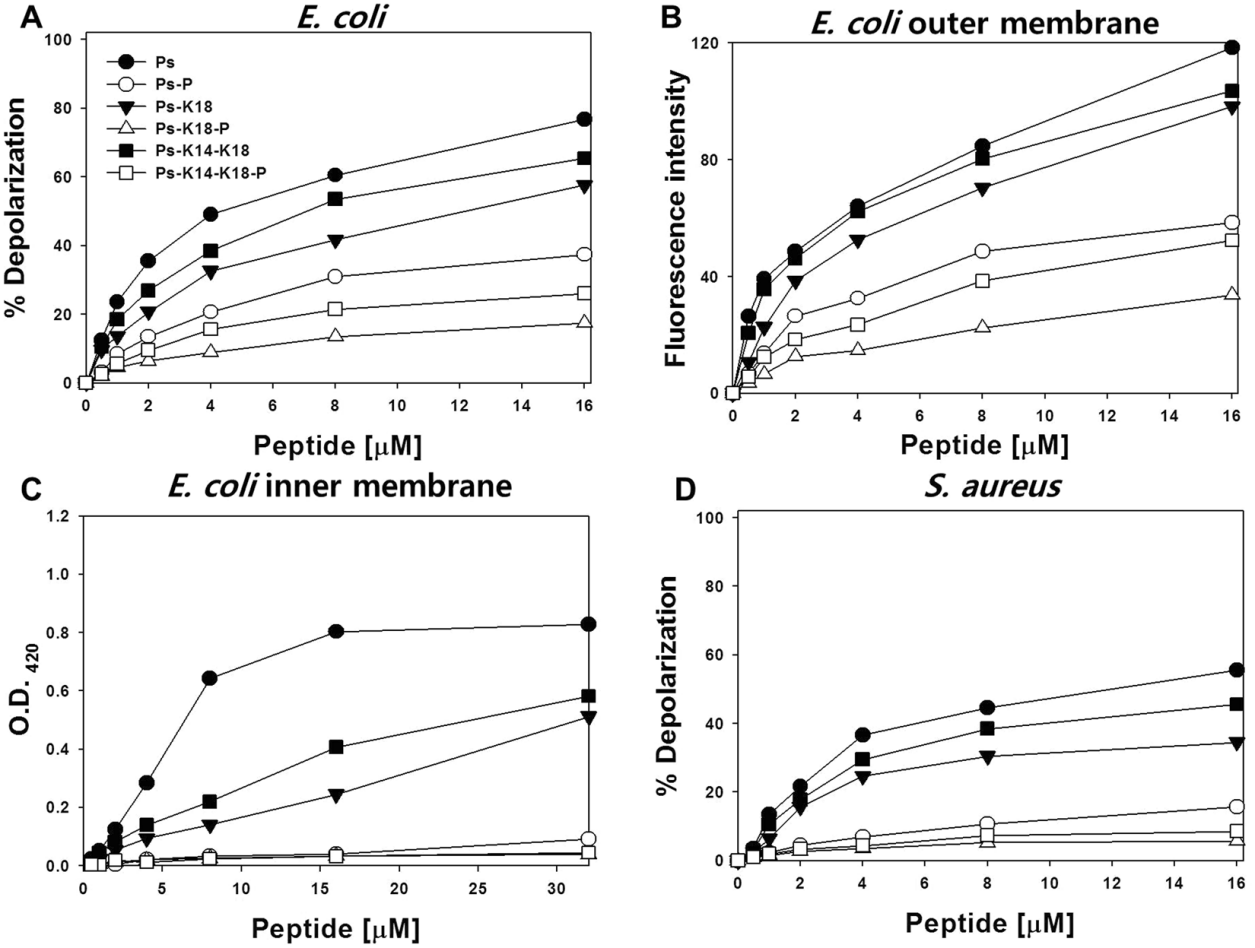
(**A**) Concentration-dependent membrane depolarization of intact *E*. *coli* KCTC 1682, monitored as an increase in diSC_3_-5 fluorescence. (**B**) Permeabilization of the outer membrane of *E*. *coli* KCTC 1682 monitored as an increase in NPN fluorescence intensity. (**C**) Permeabilization of the inner membrane of *E*. *coli* ML35p monitored as hydrolysis of ONPG by β-galactosidase. (**D**) Membrane depolarization of *S*. *aureus* KCTC 1621, monitored as an increase in diSC_3_-5 fluorescence.

Because Ps analogues exhibited high antibacterial activity against Gram-negative bacteria, we investigated the abilities of the peptides to depolarize Gram-negative bacterial membranes using *E*. *coli* (Fig. 4A). In this assay, depending on the membrane potential, diSC_3_-5 is distributed either inside the cells or in the medium, and it self-quenches when concentrated inside bacterial cells. Ps-P, Ps-K18-P and Ps-K14-K18-P produced 30–40% less depolarization of the *E*. *coli* membrane than Ps, Ps-K18 and Ps-K14-K18 at 16 μM.

Because Gram-negative bacteria have both outer and inner membranes, we tested the permeability of each membrane separately. To investigate outer membrane permeability, we used the hydrophobic probe NPN (Fig. 4B). Ps, Ps-K18 and Ps-K14-K18 induced higher amounts of NPN uptake than Ps-P, Ps-K18-P and Ps-K14-K18-P. Furthermore, inner membrane permeability was monitored by measuring the hydrolysis of o-nitrophenyl-β-D-galactoside (ONPG) (Fig. 4C). Ps-K18 and Ps-K14-K18 resulted in much lower permeability than Ps, implying that hydrophobicity is important for inner membrane permeability. All Pro-substituted analogues induced almost no inner membrane permeability. These results indicate that the mechanism of action of Ps-P, Ps-K18-P and Ps-K14-K18-P may be different from that of Ps series analogues.

To compare the Gram-positive membrane depolarization abilities of the peptides, we conducted a transmembrane depolarization assay using *S*. *aureus* (Fig. 4D). We found that Ps and its analogues produced approximately 25% less depolarization in Gram-positive *S*. *aureus* membranes than in Gram-negative *E*. *coli* membranes. All Ps-P series peptides exhibited very low membrane permeabilization abilities against *S*. *aureus*. Similar to the calcein dye leakage results in model membrane vesicles, this implies that Ps and its analogues are more effective against Gram-negative bacteria than against Gram-positive bacteria. The dye leakage assay and membrane disruption assays against *E*. *coli* and *S*. *aureus* imply that Ps-P series peptides do not target bacterial cell membranes and may penetrate bacterial cell membranes with a mechanism of action different from that of Ps series peptides.

### Inhibition of NO production in LPS-stimulated RAW264.7 cells

**Figure 5:**
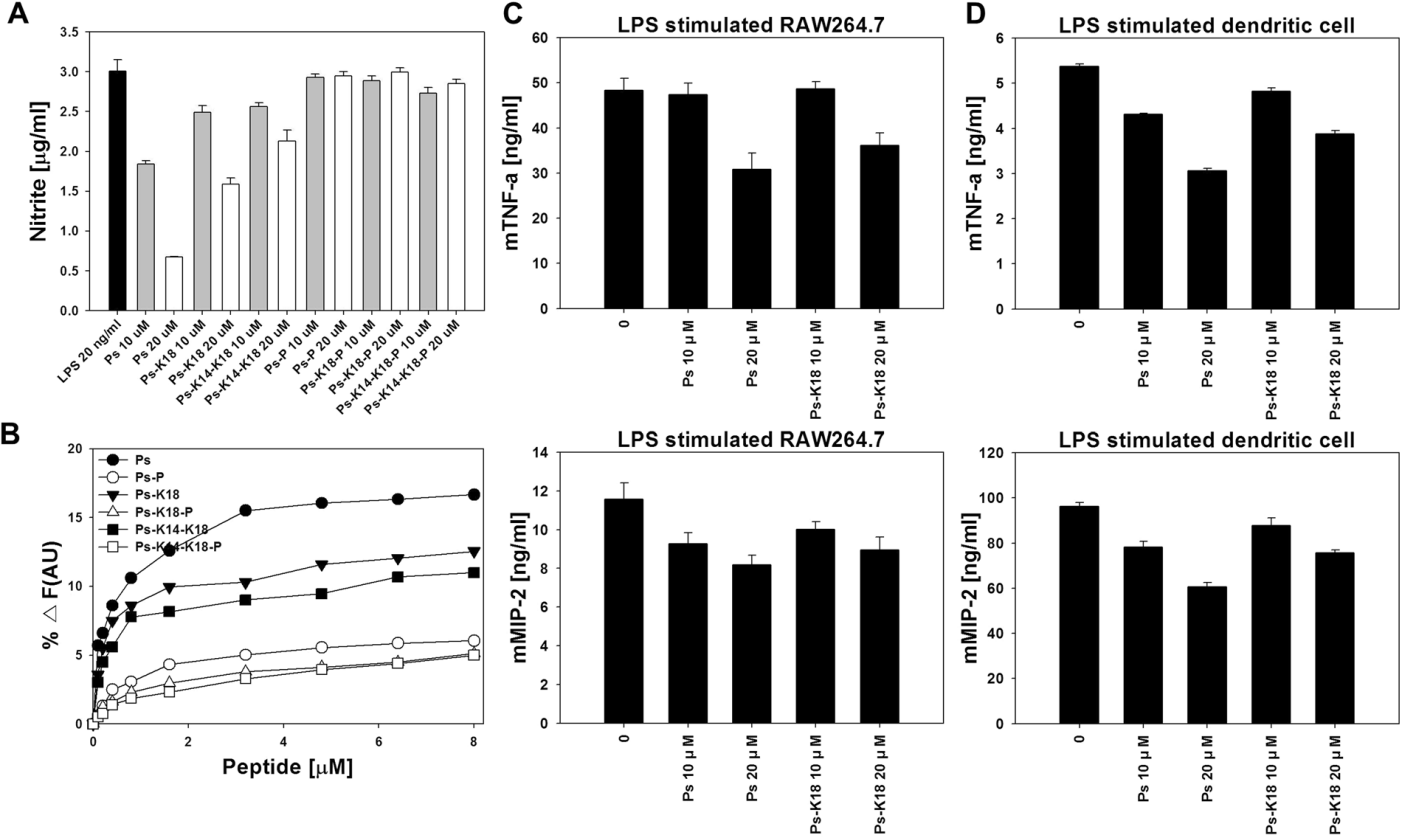
(**A**) Inhibition of nitrite production by peptides (10 μM and 20 μM) in LPS-stimulated RAW264.7 cells. (**B**) Disaggregation of LPS by the peptides. Enhancement of the intensity of FITC-labelled LPS as a function of the concentration of peptides. Effects of Ps and Ps-K18 on LPS-induced expression of inflammatory cytokines in (**C**) RAW264.7 cells and (**D**) dendritic cells as determined by enzyme-linked immunosorbent assay.

To identify the peptide features responsible for the anti-inflammatory activity of Ps, we indirectly measured peptide-induced inhibition of NO production in LPS-simulated RAW264.7 macrophages. As shown in Fig. 5A, Ps and Ps-K18 effectively inhibited NO production in LPS-stimulated RAW264.7 macrophages, with 20 μM Ps and Ps-K18 inhibiting NO production by 78% and 47%, respectively. However, 20 μM Ps-K14-K18 resulted in much lower NO inhibition. At 10 μM, Ps-K14-K18 did not inhibit NO production, whereas 10 μM Ps and Ps-K18 still showed NO inhibition, implying that optimum cationicity is important for anti-inflammatory activity. Ps-P analogues did not inhibit NO production.

### LPS neutralization of peptides

The ability of the peptides to neutralize the LPS was measured using LAL assay. All peptides showed no LPS neutralization even at 20 µM (Supplementary Fig. S4). Control peptide, LL-37 which is known as a potent anti-inflammatory peptide shows high LPS neutralization^24^. This is in agreement with the previous study, in which Ps with an amidated C-terminus did not neutralize LPS^20^.

### FITC-labelled LPS aggregates

We next examined the ability of Ps and its analogues to dissociate large LPS aggregates by monitoring increases in fluorescence using FITC-conjugated LPS^28^. Peptide addition caused a concentration-dependent increase in FITC-LPS fluorescence, indicating that interaction with Ps (or a Ps analogue) resulted in LPS dissociation. Ps, Ps-K18 and Ps-K14-K18 caused much higher increases in FITC-LPS fluorescence than Ps-P series peptides, indicating that Ps series analogues exhibit stronger interactions with LPS. The strongest interaction was observed with Ps, followed by Ps-K18, Ps-K14-K18, Ps-P, Ps-K18-P and Ps-K14-K18-P (Fig. 5B). This result is consistent with the results of the NO inhibition and antibacterial activity assays.

### Quantification of inflammatory cytokines for Ps and Ps-K18

Because Ps and Ps-K18 exhibited the highest NO inhibition and highest dissociation of LPS aggregates as well as low cytotoxicity, they were used for further analysis of anti-inflammatory activities. We investigated the mRNA expression of the cytokines mTNF-α and mMIP-2 in LPS-stimulated RAW264.7 cells. As shown in Fig. 5C, treatment with 20 μM Ps or Ps-K18 gradually suppressed the expression of the inflammation-related cytokines mTNF-α and mMIP-2 in LPS-stimulated macrophages. mTNF-α levels in Ps- andPs-K18-treated macrophages decreased by 27% and 15%, respectively, whereas mMIP-2 levels decreased by 28% and 18%, respectively.

Because dendritic cells are well-known as the antigen-presenting cells of the immune system^29^ we also examined inflammatory cytokine production in LPS-stimulated dendritic cells (Fig. 5D). mTNF-α levels in dendritic cells treated with 20 μM Ps or Ps-K18 decreased by 43% and 28%, respectively, whereas mMIP-2 levels decreased by 37% and 21%, respectively.

### Effects of Ps and Ps-K18 on LPS-stimulated pro-inflammatory cytokines and inflammatory-related proteins in RAW264.7 and dendritic cells

**Figure 6:**
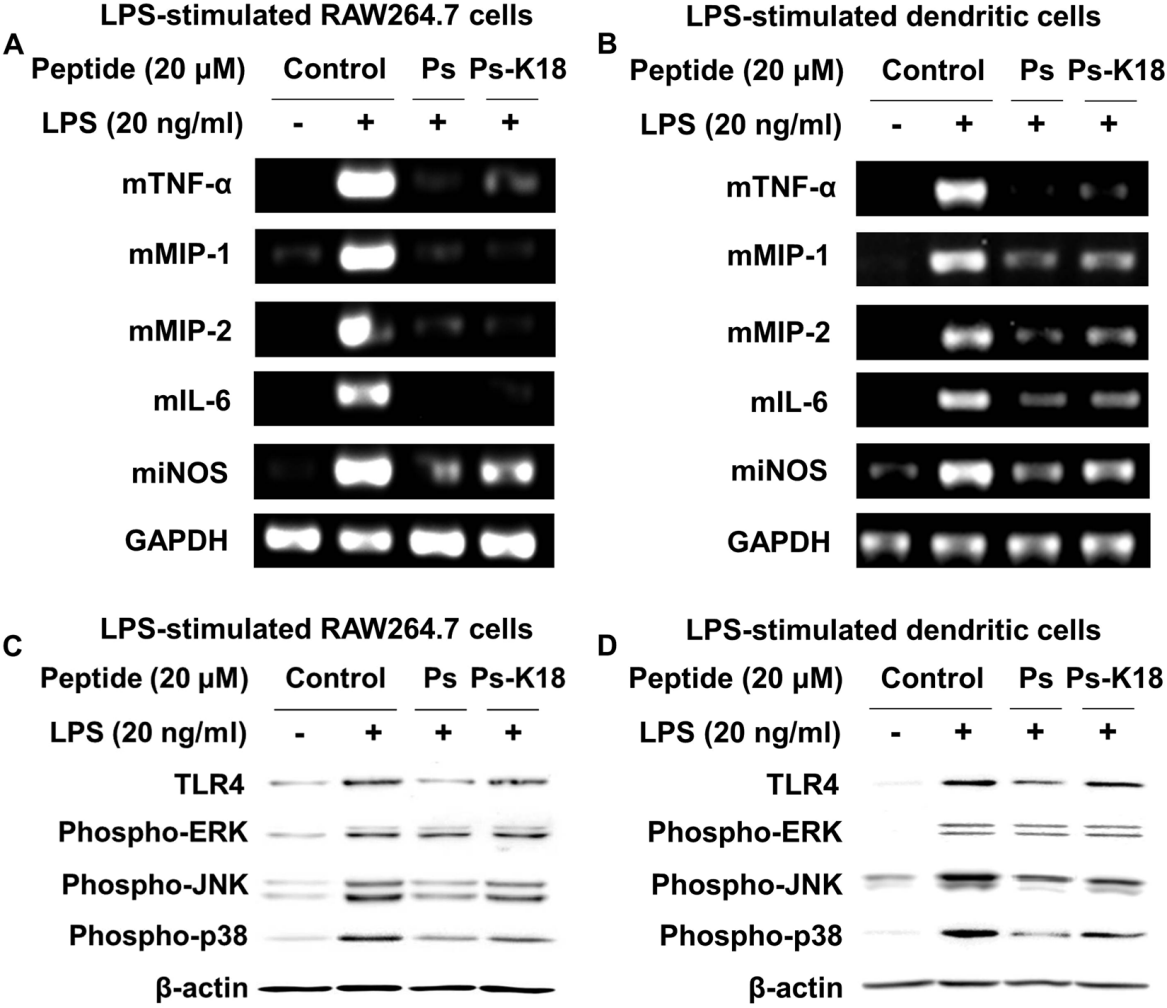
Effects of Ps and Ps-K18 on LPS-stimulated expression of pro-inflammatory cytokines in (**A**) RAW264.7 cells and (**B**) dendritic cells determined by reverse transcriptase polymerase chain reaction. Inhibition of inflammatory-related protein expression in LPS-stimulated ﻿(**C**) ﻿RAW264.7 cells and (**D**) dendritic cells.

We next elucidated the effect of Ps and Ps-K18 on LPS-stimulated inflammation in RAW264.7 and dendritic cells by measuring the expression of inflammatory cytokines using RT-PCR. Expression of mTNF-α, mMIP-1, mMIP-2, mIL-6 and miNOS was induced by 20 ng/ml LPS. Ps and Ps-K18 effectively suppressed expression of all inflammatory cytokines (Fig. 6A,B).

We also investigated the effect of Ps and Ps-K18 on suppression of LPS-stimulated expression of TLR4 and phosphorylation of MAPKs in RAW264.7 and dendritic cells using western blots (Fig. 6C,D). Ps treatment reduced the protein expression of TLR4, phospho-ERK, phospho-JNK and phospho-p38 by 73%, 0%, 58% and 58%, respectively, in RAW264.7 cells and by 57%, 10%, 62% and 74%, respectively, in dendritic cells. Ps-K18 treatment reduced the protein expression of TLR4, phospho-ERK, phospho-JNK and phospho-p38 by 16%, 7%, 23% and 20%, respectively, in RAW264.7 cells and by 15%, 8% 48%, and 44%, respectively, in dendritic cells. These results show that pre-treatment with Ps or Ps-K18 significantly inhibits LPS-stimulated expression of TLR4 and phosphorylation of JNK and p38 MAPK in RAW264.7 and dendritic cells.

### Binding of peptides with TLR4 or MD2

**Figure 7:**
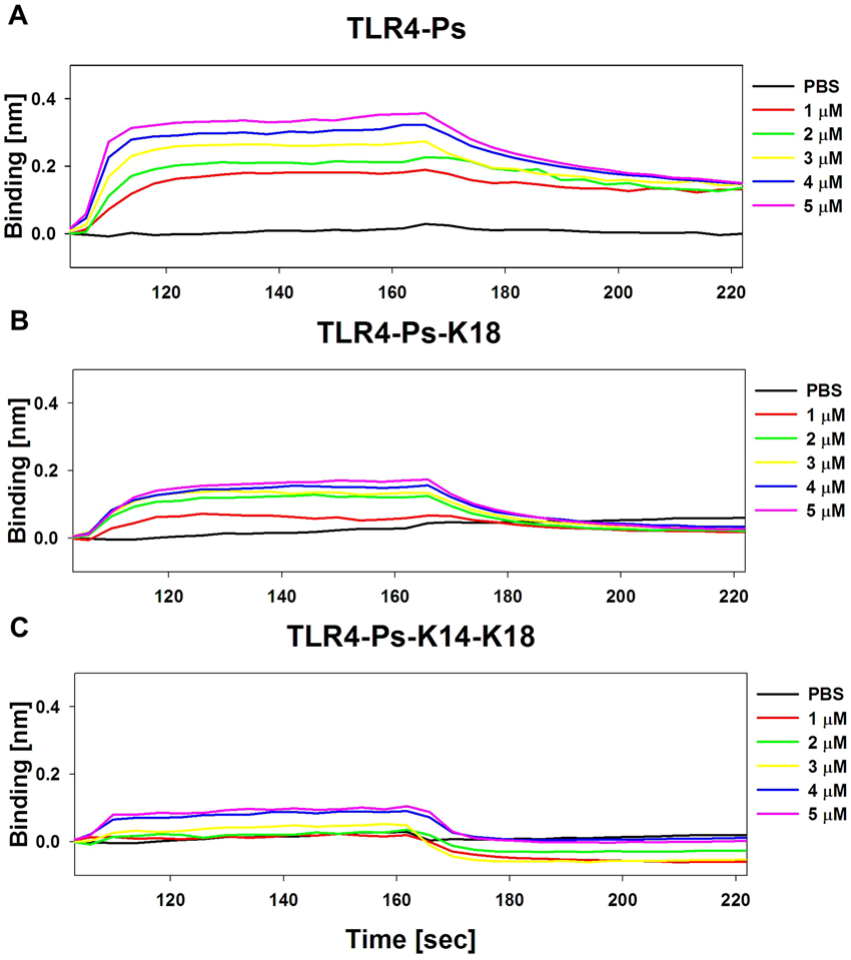
Binding between TLR4 and (**A**) Ps, (**B**) Ps-K18 and (**C**) Ps-K14-K18, as measured by bio-layer interferometry (BLI).

Because Ps and Ps-K18 decreased the level of TLR4 in LPS-stimulated RAW264.7 cells and dentritic cells but they do not neutralize LPS, we investigated the direct interaction between the peptides and TLR4 or myeloid differentiation 2 (MD2) by using bio-layer interferometry (BLI). TLR4 functions with its binding partner MD2 to recognize LPS and activate TLR4 signalling^30^. The shift of the interference pattern of a TLR4- or MD2-attached bio-sensor was monitored by applying various amounts of the peptides. The measured binding affinity for the interaction between TLR4 and Ps, Ps-K18 and Ps-K14-K18 was very high, 0.8, 1.5, and 6.9 μM, respectively (Fig. 7A–C). The binding affinity between TLR4 and Ps-P peptides was far lower than that of Ps. Both Ps and Ps-P series peptides did not bind to MD2 at all (data not shown).

## Discussion and Conclusion

Many kinds of AMPs have been isolated in the skin of various frogs from *Ascaphidae*, *Bombinatoridae*, *Hylidae*, *Hyperoliidae Myobatrachidae*, *Pipidae*, and *Ranidae* families^7–10, 12, 13, 31, 32^. Antimicrobial peptides from frog skin comprise between 12 and 48 residues and their amino acid sequences do not have any conserved domain associated with biological activity^33^. Frogs live in pathogen favorable moist and warm environments during their life cycle. Therefore, they had developed a host defense system to protect the skin from the pathogens. Those AMPs play important role in the innate immunity and defense against the pathogenic microorganisms. Most of the frog skin AMPs are cationic in charge, varying from +2 to +6, and contains high contents of hydrophobic residues. Due to higher efficacy of frog skin AMPs against a wide range of pathogens and drug resistant bacteria, considerable effort has been made to develop therapeutics from these peptides. Many efforts have been carried out to improve the antibacterial activity and reduce the cytotoxicity of these peptides by reducing their the size, altering charge, amphipathicity and hydrophobicity^14, 27, 34, 35^.

Cationicity determines interactions between AMPs and the negatively charged phospholipid membranes of bacteria^36^. Helicity also plays a crucial role in determining the activity of AMPs^37, 38^. Introducing a proline substitution is a well-known technique for disrupting helical structure in peptides^39^. One drawback of the use of AMPs in therapeutics is their cytotoxicity, arising from their inability to distinguish host cells from pathogen cells. Thus, to improve the suitability of AMPs as therapeutic agents, the cell selectivity of peptides must be improved. An optimal balance among physiochemical properties is required for increased cell selectivity and therapeutic potential^40^. In this study, we attempted to develop novel peptide antibiotics with higher selectivity for bacterial cells by synthesizing Lys-substituted and Pro-substituted analogues of Ps (Ps and Ps-P analogues).

Ps is known to have potent antimicrobial activity^14^. Park *et al*. reported that the high antimicrobial activity of Ps with an amidated C-terminus results from the formation of pores in the cytoplasmic membrane^21^. However, naturally occurring Ps has a non-amidated C-terminal end. To understand the mechanism of action and structure-activity relationships of naturally occurring Ps, we determined the 3D structures of Ps and Ps-P. These structures revealed that in DPC micelles, Ps had an amphipathic linear α-helical structure from Leu^2^ to Gln^24^ with hydrophobic side chains on one side and hydrophilic side chains on the other side, resulting in effective permeabilization of bacterial membranes^41, 42^. This implies that continuous amphipathic α-helicity is important for effective membrane disruption. In contrast, Ps-P had two α-helices and a bend at Pro^11^ in between the two helices. This Pro formed a hinge in the central region of the α-helical antimicrobial peptide, which was important for conferring high selectivity against bacterial cells and cell-penetrating ability. Compared to Ps, Ps-K18 had an increase of +1 in net charge and has similar α-helical contents in CD spectra. Ps-K18 exhibited reduced cytotoxicity while maintaining similar antimicrobial activity against Gram-negative bacteria. In addition, the bent structures of Ps-P, Ps-K18-P and Ps-K14-K18-P resulted in weaker interactions with bacterial cell membranes, with lower antimicrobial activities. The results from biological and biophysical experiments imply that Ps-K18 may be a potent candidate AMP, whereas a Pro substitution in Ps provides bacterial cell selectivity. As most of the natural peptides, Ps-K18 may have low proteolytic stability. Incorporation of unnatural amino acids such as D amino acids or peptoids in the peptide can improve the stability of Ps-K18 in human serum, and thereby make the peptides suitable for therapeutic applications.

Gram-negative bacteria have both the outer and inner membranes, whereas Gram-positive bacteria have a single cytoplasmic membrane. To examine whether Gram-negative cytoplasmic membranes are targets of Ps and its analogues, the ability of each peptide to permeabilize the outer and inner membranes of *E*. *coli* was tested using membrane depolarization experiments. Ps series peptides caused significant permeabilization of the membranes whereas Pro-substituted peptides may be able to penetrate the membrane without causing membrane damage. It was previously shown that proline-rich AMPs are able to penetrate the membrane and bind to the 70S ribosomes of bacteria^43–45^. It has also been reported that proline-rich peptides can bind to bacterial DnaK^46, 47^. Ps-P analogues may thus also act on such intracellular targets. Exact mechanism of action of Ps-P series peptides should be investigated in future.

We also investigated the anti-inflammatory activities of Ps and its analogues. Among all peptides tested, only Ps and Ps-K18 significantly inhibited NO production in LPS-induced RAW264.7 cells. Since none of the Ps analogues showed LPS neutralization from the LAL assay, we investigated the dissociation of LPS aggregates by Ps analogues to understand the mode of anti-inflammatory activity. Similar to the NO inhibition, Ps and Ps-K18 dissociated LPS aggregates effectively. Because Ps has a net charge of +2 and Ps-K18 has a net charge of only +3, they have rather weak electrostatic interactions with LPS compared to other AMPs. Despite its higher antibacterial and depolarizing activities against *E*. *coli*, Ps-K14-K18 with a net charge of +5 showed only poor anti-inflammatory activity. These results imply that the major interactions of Ps or Ps-K18 with LPS may not be mediated through electrostatic interactions between the polar polysaccharide part of LPS and the peptides, but rather through the hydrophobic interactions between the phospholipid part of lipid A and the peptides. Therefore, hydrophobicity and linear alpha-helical structures, not electrostatic interactions, are the key factors for the anti-inflammatory activity of the peptides.

Even though the peptides did not neutralize LPS directly, they inhibited NO formation and dissociate LPS aggregates. Therefore, we measured the direct binding of the peptides to TLR4 and MD2 using bio-layer interferometry. Ps, Ps-K18 and Ps-K14-K18 peptide bind to TLR4 binds tightly while the binding affinity between TLR4 and Ps-P peptides was very low. These results imply that a linear α-helical structure is essential for the interaction of the peptides with TLR4. In contrast, all of the peptides did not bind to MD2. Rakhesh *et al*. reported that the SPA4 peptide had a mechanism of anti-inflammatory activity similar to that of Ps, where the peptide did not bind to LPS, but rather bound to TLR4 and inhibited TLR4-NFκB signalling^48^. It has been reported that lipid chains of LPS binds to the hydrophobic pocket of MD2 in the TLR4/MD2 complex, facilitating the formation of a multimer composed of two copies of the TLR4-MD-2_LPS symmetric complex^30^, leading to activation of inflammation pathways. The observed anti-inflammatory activities of Ps and Ps-K18 may arise from inhibition of the formation of the TLR4-MD-2_LPS complex by direct binding of the peptides to TLR4. Western blot results revealed that Ps and Ps-K18 regulated the expression of TLR4 and the phosphorylation of JNK and p38 MAPK in LPS-stimulated RAW264.7 and dendritic cells.

The structures of Ps and Ps-P determined in DPC micelle are the structures in membrane mimetic environment. If we assume that these structures of peptides in DPC micelles are similar to the structures in the bacterial cell membrane, we can propose that increasing the amphipathicity of the structure by reducing the hydrophobicity and increasing the cationicity as well as introducing a bend in the structure may be critical for improving bacterial cell selectivity. The linear α-helical structures of Ps and Ps-K18 with an optimal balance between hydrophobicity and cationicity may be crucial for their anti-inflammatory activity as well as for the dissociation of the lipid A part of LPS aggregates. Direct binding of these peptides to TLR4 may result in inhibition of the formation of the TLR4-MD-2_LPS complex. The flexible bend structure induced by Pro in Ps-P analogues may be important for facilitating penetration of the bacterial cell membrane and conferring bacterial cell selectivity to Ps-P series peptides. This study can provide insight into the design of potent AMPs with anti-inflammatory activity and bacterial cell selectivity.

## Methods

### Peptide Synthesis

All six peptides listed in Table 1 were prepared by solid-phase synthesis using Fmoc chemistry. Peptides were purified by reversed-phase preparative high-performance liquid chromatography on Vydec C_18_ column (4.6 × 250 mm) using a gradient of 5% to 95% acetonitrile in H_2_O with 0.05% TFA delivered for 30 min. Peptide purities were higher than 95%. The peptides mass determined by matrix-assisted laser desorption/ionization time-of-flight mass spectrometry as well as retention times are listed in Table 1.

### Antimicrobial activity

Four Gram-negative bacteria species (*Escherichia coli* KCTC 1682, *Acinetobacter baumannii* KCTC 2508, *Pseudomonas aeruginosa* KCTC 1637 and *Salmonella typhimurium* KCTC 1926) and three Gram-positive bacteria species (*Staphylococcus aureus* KCTC 1621, *Bacillus subtilis* KCTC 3028 and *Staphylococcus epidermidis* KCTC 1917) were procured from the Korean Collection for Type Cultures (KCTC). Multi-drug-resistant Gram-negative bacteria (*Salmonella typhimurium* CCARM 8003 and 8007, *Escherichia coli* CCARM 1229 and 1238, *Pseudomonas aeruginosa* CCARM 2002 and 2095 and *Acinetobacter baumannii* CCARM 12035 and 12036) were obtained from the Culture Collection of Antibiotic-Resistant Microbes (CCARM). To determine MICs, bacteria were cultured in Luria-Bertani (LB) medium for overnight at 37 °C. An aliquot of the culture was incubated in 1% peptone media at 37 °C until mid-log phase. 100 μl of serial 2-fold diluted peptides were treated in 96 well plates and added to 100 μl of 2 × 10^6^ CFU/ml bacterial suspensions in 1% peptone media for 16 h at 37 °C. The lowest concentration of peptide that completely inhibited bacterial growth was determined to be the MIC.

### NMR analysis and structure calculation

Peptides (1.0 mM) were dissolved in 450 μl of 9:1 H_2_O/D_2_O(w/w) containing 200 mM DPC micelles. Phase-sensitive two-dimensional experiments, including nuclear NOESY, TOCSY and DQF-COSY, were performed on a Bruker 700 and 800 MHz spectrometer at the Korea Basic Science Institute (Ochang, Korea) as previously described^49, 50^. Structure calculations of the peptides were performed by following the standard protocol of the Cyana3.9 program in Linux^51^. We obtained 100 structures, and the 20 with the lowest energies were selected for each peptide (PDB accession 2NCX for Ps and 2NCY for Ps-P).

### Haemolytic activity

Peptide-induced lysis of human red blood cells (hRBCs) was determined as follows. Fresh hRBCs were washed 2–3 times with phosphate-buffered saline (PBS) by centrifugation for 5 min at 2500 rpm at 4 °C. Peptides in PBS were incubated with 4% (v/v) hRBCs for 1 h at 37 °C. Samples were then centrifuged for 5 min at 1000 × *g*. The absorbance of the supernatant at 405 nm was used as a measure of haemolysis. As a positive control, 100% haemolysis was obtained by treating hRBCs with 0.1% Triton X-100.

### Cytotoxicity

The MTT assay was used to determine the cytotoxicity of each peptide against mouse macrophage RAW264.7 cells and mouse bone marrow-derived dendritic cells. Bone marrow was isolated from 6-week-old female C57BL mice. Red blood cell lysis buffer (Sigma) was used to lyse red blood cells in the bone marrow. Then, the bone marrow was cultured in RPMI 1640 containing 10% foetal bovine serum and 20 ng/ml GM-CSF. After three days, floating cells were gently removed, and fresh medium was added. Dendritic cells were extracted three days later. Cells were cultured on 96-well plates at a density of 1 × 10^4^ cells/well in RPMI 1640 containing 10% foetal bovine serum. Different concentrations of peptides were added and incubated for 18 h at 37 °C. Subsequently, 20 μl of MTT (5 mg/ml) was added to each well and then incubated for 4 h at 37 °C. An ELISA reader was used to measure the absorbance at 570 nm. A 100% cytotoxic control was generated by treatment with 0.1% Triton X-100. All experiments and protocols for animal care were reviewed and approved by the Institutional Animal Care and Use Committee of Konkuk University (KU15046).

### Calcein dye leakage and Membrane disruption assay

Calcein-entrapped LUVs with three different lipid compositions were prepared as described previously^45^. The percentage of calcein that had leaked from the membranes after 3 min of exposure to the peptide was used as a measure of membrane permeability^45^. The membrane depolarization activity of each peptide was determined using *E*. *coli* and *S*. *aureus* as described previously^50^. Outer membrane permeability was measured using NPN dye and *E*. *coli* cells. An ONPG hydrolysis assay was performed to measure inner membrane permeability of *E*. *coli* ML-35p cells as described previously^50^.

### Anti-inflammatory activity

The amount of nitrite in cell culture medium was used for detection of nitric oxide (NO). RAW264.7 cells were incubated in 96-well plates at a density of 1 × 10^5^ cells/well and stimulated with 20 ng/ml LPS in the presence of each peptide for 18 h. The supernatant was mixed with an equal volume of 4% Griess reagent. The absorbance was measured at 540 nm. To quantify inflammatory cytokine levels, RAW264.7 cells or dendritic cells were treated as in the NO assay. The supernatants, in two-fold serial dilutions, were added to antibody capture pre-coated plates for 2 h at 25 °C. Inhibition of inflammatory cytokine release was determined using a sandwich ELISA assay as previously described^52^. To measure peptide-induced inhibition of expression of inflammatory cytokine mRNA and inflammation-related proteins, we performed reverse transcription polymerase chain reaction (RT-PCR) and western blot assays as previously described^25, 49^.

### Interaction of peptides with LPS and TLR4

LPS neutralization by the peptides were measured using LAL-QCL-1000^TM^ (Lonza, USA) kit using a modified protocol as described previously^21^. The interaction between each peptide and FITC-LPS was monitored according to the change in fluorescence intensity (excitation = 480 nm, emission = 515 nm). Peptides and 0.5 μg/ml FITC-LPS were prepared in 10 mM phosphate buffer (pH 6.0). The binding between rhTLR4 (from R&D systems, Minneapolis, USA) or rhMD2 (R&D systems, Minneapolis, USA) and peptides was determined using the BLItz system (ForteBio, Menlo Park, CA)^53^. A Ni-NTA sensor was used to detect rhTLR4 and rhMD2 binding. Before the binding assay, the biosensor was hydrated for 10 min in PBS. An initial baseline of 15 s, loading for 60 s, baseline for 30 s, association for 60 s and dissociation for 60 s were used for measurement.

## Electronic supplementary material

Supplementary information
