# Supplementary material for: Investigation of cationicity and structure of pseudin-2 analogues for enhanced bacterial selectivity and anti-inflammatory activity
Source: Sci Rep. 2017 May 3;7:1455. doi: 10.1038/s41598-017-01474-0 (PMC5431190; doi:10.1038/s41598-017-01474-0)
Supplement: Supplementary file 1 — Supplementary information [file 41598_2017_1474_MOESM1_ESM.doc]

**Supplementary Information**

**Investigation of cationicity and structure of pseudin-2 analogues for enhanced bacterial selectivity and anti-inflammatory activity**

Dasom Jeon^a^, Min-Cheol Jeong^a^, Binu Jacob^a^, Jeongkyu-Bang^b^, Eun-Hee Kim^b^, Chaejoon Cheong^b^, In Duk Jung^c^, Yoon Kyung Park^d^, and Yangmee Kim^a^,*

*^a^Department of Bioscience and Biotechnology, Konkuk University, Seoul 05029, Korea*

*^b^Division of Magnetic Resonance, Korea Basic Science Institute, Ochang,* ***28119****, Korea*

*^c^Department of Immunology, Lab of Dendritic Cell Differentiation & Regulation, School of Medicine, Konkuk University, Chungju,380-701, Korea*

*^d^ Department of Biomedical Science and Research Center for Proteinaceous Materials (RCPM), Chosun University, Gwangju* ***61452****, Korea*

^*^ Corresponding author: Yangmee Kim, Ph. D.

Department of Bioscience and Biotechnology, Konkuk University, Seoul 143-701, South Korea. Tel.: +822-450-3421; Fax: +822-447-5987; E-mail address: ymkim@konkuk.ac.kr

**
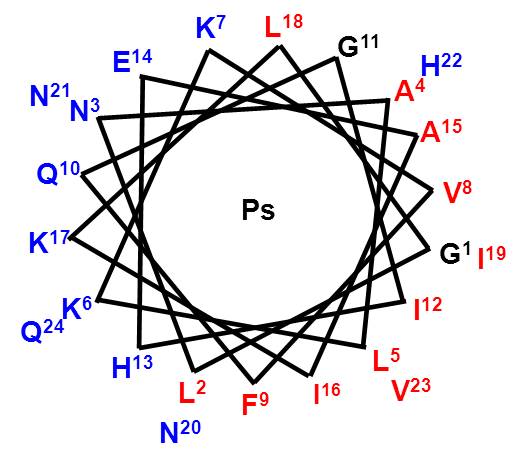
**

**FIGURE S1.** α-helical wheel diagram for Ps. The hydrophobic residues are indicated in red, and the hydrophilic residues are shown in blue.


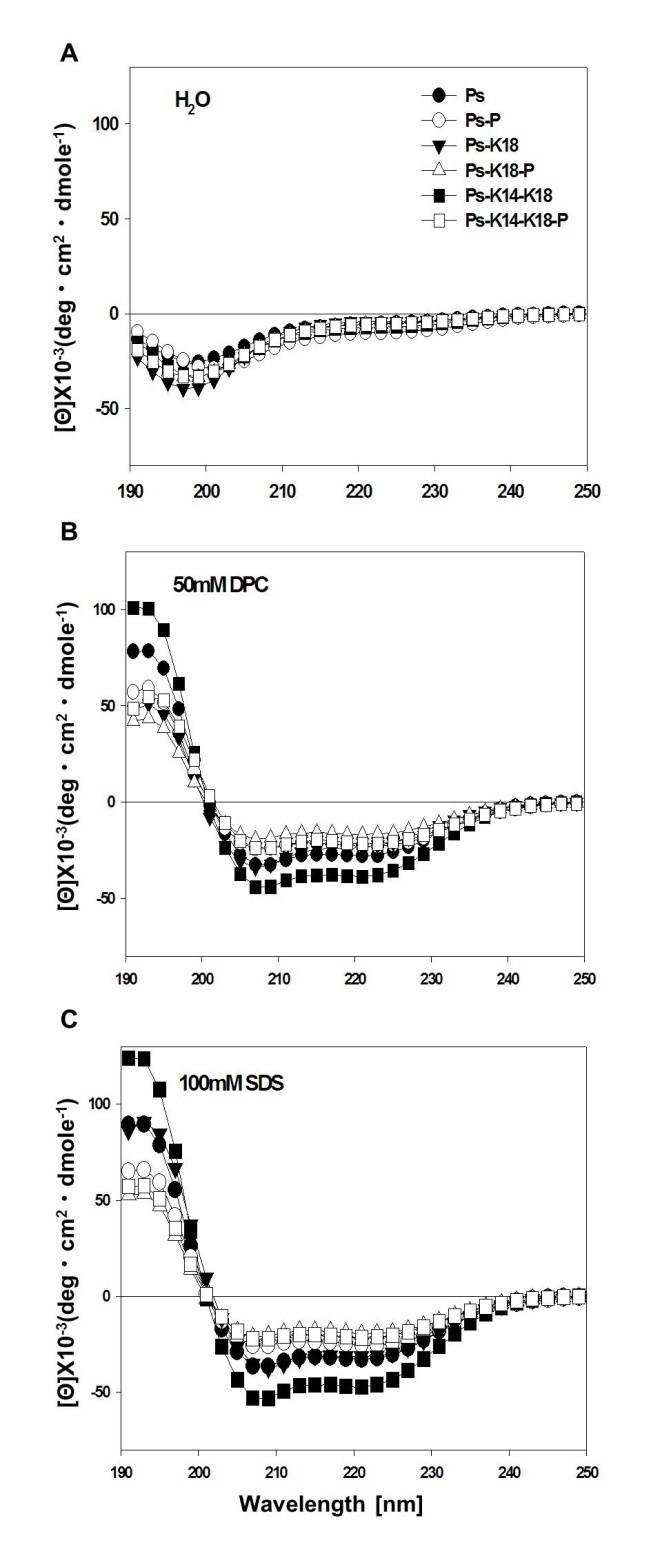


**FIGURE S2.** CD spectra of the peptides in (A) H_2_O, (B) 50 mM dodecylphosphocholine (DPC) micelles and (C) 100 mM SDS micelles.

**
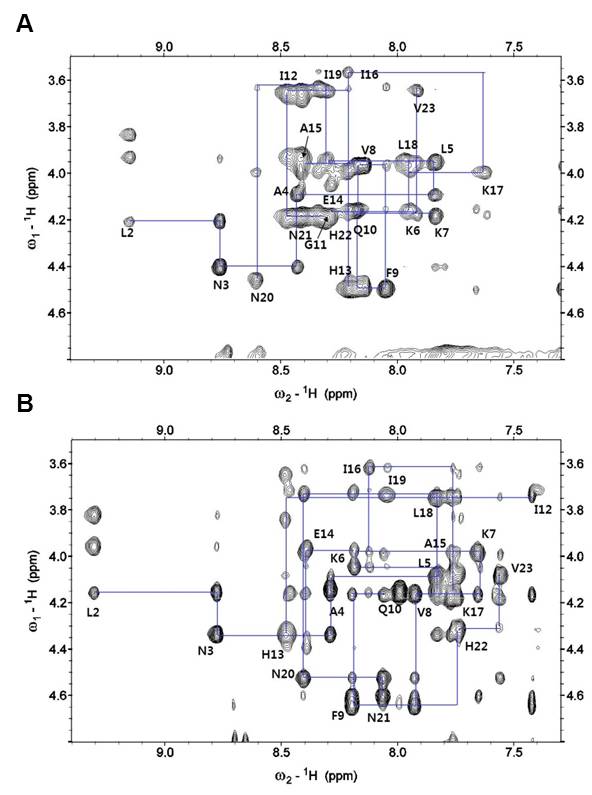
**

**Figure S3.** NOESY spectra of the NH-Cα H region of (A) Ps and (B) Ps-P in 200 mM DPC micelles at 303K.


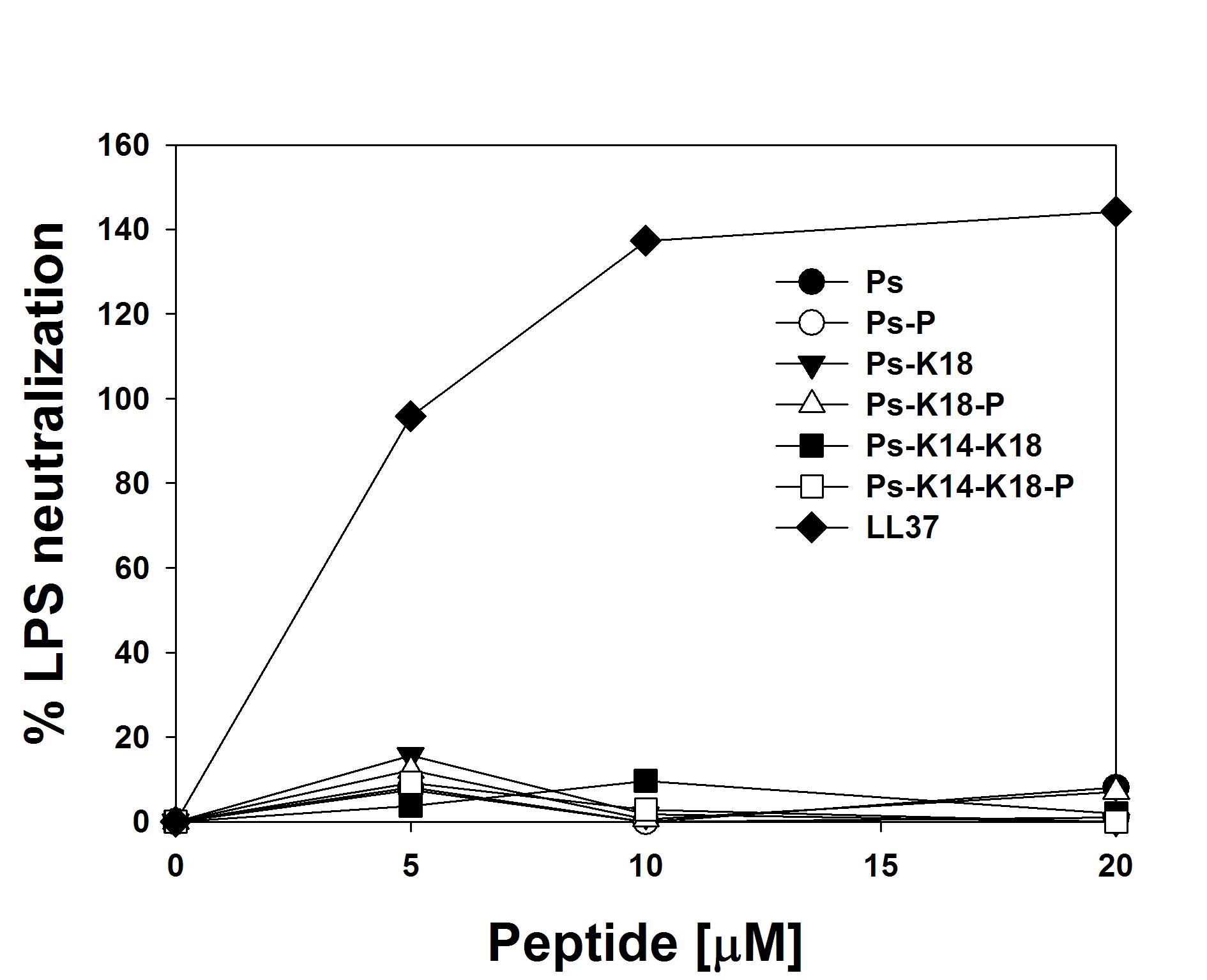


**Figure S4.** The ability of the peptides to neutralize the LPS .

**Table S1.** Structural statistics and mean pairwise root mean squared deviations (RMSD) for the 20 lowest-energy structures of Ps and Ps-P in 200 mM DPC micelles at 303K.

|  | Ps | Ps-P |
| --- | --- | --- |
| **Distance restraints** |  |  |
| Short-range | 174 | 127 |
| Medium-range | 43 | 53 |
| Long-range | 0 | 9 |
| Total | 217 | 189 |
| H-bond | 18 | 14 |
| Mean Cyana target function (Å^2^) | 0.05± 0.01 | 0.03± 0.01 |
| **Deviation from mean structure** |  |  |
|  | Residues 2-24 | |
| Backbone atoms | 0.18 ± 0.06 | 0.34 ± 0.13 |
| Heavy atoms | 0.77 ± 0.08 | 0.80 ± 0.09 |
| **Ramachandran plot for the mean structure** |  |  |
| Residues in the most favourable allowed region (%) | 99 | 98.8 |
| Residues in the additionally allowed region (%) | 1 | 1.2 |
| Residues in the disallowed region (%) | 0 | 0 |
